# Composite of MIL-101(Cr) with a Pyrrolidinium-Based Ionic Liquid Providing High CO_2_ Selectivity

**DOI:** 10.1021/acsaenm.3c00010

**Published:** 2023-05-22

**Authors:** Nitasha Habib, Ozce Durak, Hasan Can Gulbalkan, Ahmet Safa Aydogdu, Seda Keskin, Alper Uzun

**Affiliations:** † Department of Chemical and Biological Engineering, Koç University, Rumelifeneri Yolu Sariyer, Istanbul 34450, Turkey; ‡ Koç University TÜPRAŞ Energy Center (KUTEM), Koç University, Rumelifeneri Yolu Sariyer, Istanbul 34450, Turkey; § Koç University Surface Science and Technology Center (KUYTAM), Koç University, Rumelifeneri Yolu Sariyer, Istanbul 34450, Turkey

**Keywords:** CO_2_ separation, ionic liquid (IL), metal organic framework (MOF), IL/MOF composite

## Abstract

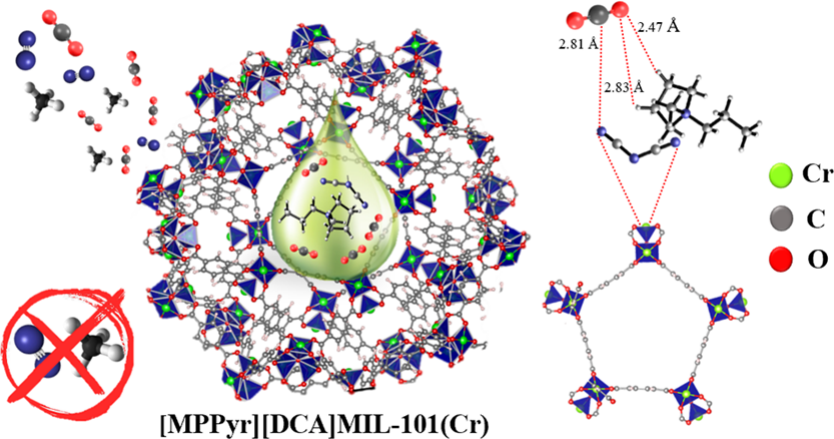

Capturing CO_2_ selectively from flue gas and natural gas addresses the criteria of a sustainable society. In this work, we incorporated an ionic liquid (IL) (1-methyl-1-propyl pyrrolidinium dicyanamide, [MPPyr][DCA]) into a metal organic framework (MOF), MIL-101(Cr), by wet impregnation and characterized the resulting [MPPyr][DCA]/MIL-101(Cr) composite in deep detail to identify the interactions between [MPPyr][DCA] molecules and MIL-101(Cr). Consequences of these interactions on the CO_2_/N_2_, CO_2_/CH_4_, and CH_4_/N_2_ separation performance of the composite were examined by volumetric gas adsorption measurements complemented by the density functional theory (DFT) calculations. Results showed that the composite offers remarkably high CO_2_/N_2_ and CH_4_/N_2_ selectivities of 19,180 and 1915 at 0.1 bar and 15 °C corresponding to 1144- and 510-times improvements, respectively, as compared to the corresponding selectivities of pristine MIL-101(Cr). At low pressures, these selectivities reached practically infinity, making the composite completely CO_2_-selective over CH_4_ and N_2_. The CO_2_/CH_4_ selectivity was improved from 4.6 to 11.7 at 15 °C and 0.001 bar, yielding a 2.5-times improvement, attributed to the high affinity of [MPPyr][DCA] toward CO_2_, validated by the DFT calculations. These results offer broad opportunities for the design of composites where ILs are incorporated into the pores of MOFs for high performance gas separation applications to address the environmental challenges.

## 1 Introduction

Carbon dioxide concentration in the atmosphere, reaching 420 ppm in June 2022,^1^ is the root cause of climate change. CO_2_ separation from flue gas and natural gas is considered as a viable solution to mitigate CO_2_ emissions.^2,3^ To selectively capture CO_2_ and purify natural gas, various applications, such as adsorption, absorption, and membrane-based separation, have been considered.^4−9^ Adsorption-based gas separation processes that utilize porous materials have been promising owing to their low energy demand and cost-effectiveness. Therefore, design and development of novel porous materials with an ability to selectively capture CO_2_ from CH_4_ and N_2_ are critical. In this regard, several kinds of adsorbents exemplified by metal organic frameworks (MOFs),^10^ zeolites,^11^ graphene-based materials,^12^ porous carbons,^13^ activated carbons,^14^ and carbon nanotubes (CNTs)^15^ have been considered. Among these, MOFs have emerged as promising materials for capturing CO_2_ from gas mixtures containing CH_4_ and N_2_.^16−18^ They are crystalline materials having high surface areas, large porosity, and good mechanical, chemical, and thermal stabilities.^19^ The physicochemical properties of MOFs can be easily tuned by changing the organic linker–metal node combination.^20^

Recent studies demonstrated that postsynthesis modification of MOFs by incorporating ionic liquids (ILs), molten salts having high thermal stability and nonvolatility with high affinity for CO_2_,^21,22^ into their pores leads to IL/MOF composites offering higher gas separation performance compared to pristine MOFs.^23−27^ The IL/MOF composites with imidazolium-based cations in the IL have been extensively studied for CO_2_/N_2_, CO_2_/CH_4_, and CH_4_/N_2_ separations.^27−32^ For example, in one of the earliest studies,^29^ 1-*n*-butyl-3-methylimidazolium tetrafluoroborate, [BMIM][BF_4_], was incorporated into the cages of CuBTC to synthesize an IL/CuBTC composite. The resulting composite provided approximately 1.5-times higher ideal selectivities for CH_4_/H_2_ and CH_4_/N_2_ compared to the pristine CuBTC at 0.2 bar. A few studies incorporated amine-functionalized ILs and polymerized ILs (PILs) into MOFs. For instance, NH_2_-MIL-101(Cr) was impregnated with an amine-functionalized *n*-aminopropyl-3-butylimidazolium bis(trifluoromethylsulfonyl)imide ([C_3_NH_2_bim][Tf_2_N]) to obtain a novel IL/MOF composite. The gas adsorption measurements indicated that IL incorporation almost doubled the CO_2_/N_2_ selectivity. This improvement was claimed to be associated with the strong Lewis acid–base and dipole–dipole interactions between CO_2_ molecules and the amine functional group.^30^ Similarly, 1-vinyl-3-ethylimidazolium bromide (poly[VEIM][Br]) was confined in MIL-101(Cr) via in situ polymerization and the resulting composite offered an improved CO_2_ uptake of 62 cm^3^/g compared to the uptake of pristine MIL-101(Cr) (57 cm^3^/g).^25^

We recently applied this concept to a rarely used family of ILs and demonstrated that the pyrrolidinium-based ILs could be very promising in boosting the CO_2_ separation performance of UiO-66.^33^ 1-*n*-Butyl-1-methylpyrrolidinium dicyanamide, [BMPyrr][DCA]-incorporated UiO-66 exhibited improved CO_2_/N_2_ and CH_4_/N_2_ selectivities (>100,000) at low pressures compared to pristine UiO-66. The high potential of this composite as an adsorbent was attributed to the exceptionally strong CO_2_-phillic characteristic of the [DCA]^−^ anion and the superior gas separation properties and low toxicity of the pyrrolidinium cations as compared to the imidazolium-based ILs.^34^ Motivated by these promising results, in this work, we used an IL having the same anion but with a different cation, 1-methyl-1-propyl pyrrolidinium dicyanamide, [MPPyr][DCA], and incorporated it into chromium(III) terephthalate MIL-101(Cr), a MOF that has a significantly high surface area and unsaturated Cr(III) sites through which CO_2_ can have strong Lewis acid–base interactions.^35,36^ [MPPyr][DCA] was selected because it was identified as an IL having high CO_2_ separation performance based on the quantum chemical equilibrium thermodynamic calculations predicting the gas solubilities in ILs.^33^ MIL-101(Cr) and [MPPyr][DCA]/MIL-101(Cr) composites were deeply characterized to identify the interactions between the IL and the MOF. The volumetric gas adsorption method was used to obtain single-component adsorption isotherms of CO_2_, N_2_, and CH_4_ at 15, 25, and 35 °C, up to 1 bar to assess the consequences of interactions between the IL and the MOF on the gas separation performance of the composite. The ideal selectivities demonstrated that the new composite has remarkably high CO_2_/N_2_ and CO_2_/CH_4_ selectivities, especially at low pressures (between 0.001 and 0.1 bar).

## 2 Experimental Methods

### 2.1 Materials

MIL-101(Cr), [MPPyr][DCA] (>98%), acetone, and the gases (CO_2_ (99.9 vol %), CH_4_ (99.999 vol %), and N_2_ (99.998 vol %)) were purchased from Nanoshel, IoLiTec, Sigma-Aldrich, and Air Liquide, respectively.

### 2.2 Synthesis of the [MPPyr][DCA]/MIL-101(Cr) Composite

For this specific composite, the IL loading to reach the wetness limit of MIL-101(Cr) was determined as approximately 45 wt %. Therefore, we set the IL loading in the composite slightly below this wetness limit as 40 wt % to ensure that the IL molecules completely remain inside the pores. The [MPPyr][DCA]/MIL-101(Cr) composite was synthesized via the wet impregnation method at a stoichiometric IL loading of 40 wt %. Prior to IL incorporation, the as-received MOF was treated in a vacuum oven at 150 °C. For the synthesis of the IL/MOF composite, first, 0.4 g of IL was combined with 20 mL of acetone and continuously stirred for 1 h until a homogeneous solution was obtained. Afterward, 0.6 g of MIL-101(Cr) was added into the solution and left for stirring at 35 °C with an open lid to allow slow evaporation of the solvent. Following the complete solvent evaporation, the composite was further dried in an oven at 125 °C overnight to remove the residual solvent. Finally, the composite was stored in a desiccator.

### 2.3 Characterization Techniques

The IL loading in the composite was determined by a measurement for which the IL/MOF composite was washed with acetone solvent to extract all the IL present in the composite. The infrared (IR) analysis of the composite was performed before and after washing the sample with acetone. The washed [MPPyr][DCA]/MIL-101(Cr) composite was then dried in an oven at 65 °C and weighed, and IL loading was determined. X-ray diffraction (XRD) analysis of the as-received MIL-101(Cr) and its composite with [MPPyr][DCA] was performed on a Bruker D2 Phaser instrument. IR spectra of the samples were collected in transmittance mode at a spectral resolution of 2 cm^–1^ in the range of 400 to 4000 cm^–1^ using a Bruker Vertex 80v spectrometer. IR peaks were deconvoluted by employing Fityk software using the Voigt function.^37^ N_2_ adsorption isotherms were measured at −196 °C, from 10^–6^ to 1 bar, by using a Micromeritics ASAP 2020 accelerated surface area and porosity analyzer. Prior to these measurements, the as-received MIL-101(Cr) and its composite with [MPPyr][DCA] were degassed at 150 °C under vacuum for 12 h. Scanning electron microscopy (SEM) images of the samples were obtained by using a Zeiss Evo LS 15 electron microscope. Thermogravimetric analysis (TGA) was done on a TA Instruments Q500 analyzer. The details of these measurements can be found in our previous report.^38^

### 2.4 Gas Adsorption Measurements

A Quantachrome volumetric gas sorption analyzer, iSorb HP2, was used to measure the single-component adsorption isotherms of CO_2_, CH_4_, and N_2_ on the samples. The sample amount was set to approximately 0.45 g for each measurement. Prior to the measurements, the samples were degassed at 125 °C under vacuum for 12 h. Gas adsorption isotherms were measured at 15, 25, and 35 °C from 0.001 to 1 bar. Repetitive measurements following the regeneration after the gas desorption step indicated that the adsorption data are reproducible within <±3% error at 1 bar. Each isotherm was then fitted to the dual-site Langmuir (DSL) and dual-site Langmuir–Freundlich (DSLF) models by using Ideal Adsorbed Solution Theory (IAST)^++^ software^39^ and the fitting parameters are provided in Table S1 of the Supporting Information (SI).

### 2.5 Computational Methodology

Solubilities of the guest gases in bulk IL were estimated by conductor-like screening model for realistic solvents (COSMO-RS) calculations by COSMOThermX software (version: C30_160) using respective triple-z valence polarized basis set (TZVP-FINE) parameterizations. More details about these calculations can be found in the literature.^40−42^ Density functional theory (DFT) calculations were performed at a level of Becke-three-parameter-Lee-Yang-Parr (B3LYP),^43,44^ including Grimme’s D2 correction,^45^ and using the 6-311 + G* basis set on Gaussian09 to evaluate the interactions between CO_2_, CH_4_, N_2_, and [MPPyr][DCA]. The electrostatic interactions were further evaluated by natural bond orbital (NBO) analyses.^46^

## 3 Results and Discussion

### 3.1 Characterization of MIL-101(Cr) and [MPPyr][DCA]/MIL-101(Cr)

**Figure 1:**
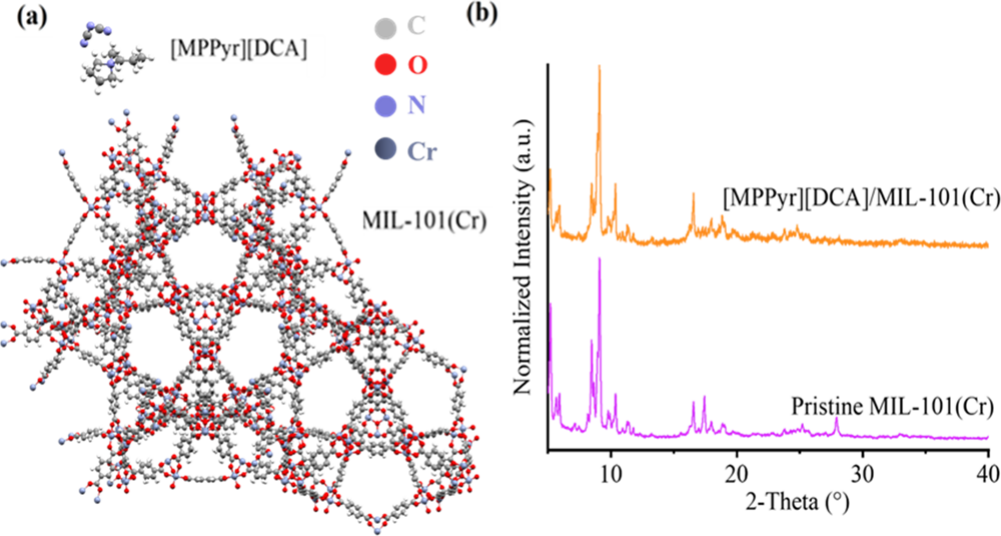
(a) Ball and stick representation of MIL-101(Cr) (snapshot taken from the a* direction) and [MPPyr][DCA]. Gray, white, blue, red, and dark gray spheres represent the C, H, N, O, and Cr atoms, respectively. (b) XRD diffractogram of the [MPPyr][DCA]/MIL-101(Cr) composite having an IL loading of 38 wt %. The XRD data for pristine MIL-101(Cr) were taken from our previous work^47^ and used for comparison.

Figure 1a provides the ball and stick representations of [MPPyr][DCA] and MIL-101(Cr). Due to the lack of any distinguishable elements in the IL (containing only C, H, N, and O, which are also present in the MOF), we could not perform X-ray fluorescence (XRF) spectroscopy measurements to quantify the corresponding IL loading in [MPPyr][DCA]/MIL-101(Cr). Instead, we washed the composite in acetone, which is small enough to enter into the pores of MIL-101(Cr), to extract any IL present in the pores of the MOF. The IR spectra shown in Figure S1 demonstrate the lack of any IL-related IR features on the washed-and-dried-composite and confirm the complete removal of the IL. By comparing the mass of the composite before and after this washing process, the IL loading was estimated to be 38 wt %, consistent with the amount of IL used for the synthesis of the composite. Here, we note that this IL loading is comfortably lower than the one used in a recent study presenting a mixed matrix membrane (MMM) having a different [MPPyr][DCA]/MIL-101(Cr) composite with a targeted IL loading of 45 wt % as the filler.^47^ Results showed that at such an excessive IL loading, some of the IL molecules remain deposited on the external surface of the MOF, and they can readily leach out during the synthesis of the MMM.^47^ Hence, having a lower IL loading of 38 wt % eliminates this possibility and leads to a completely different IL/MOF composite, where all the IL molecules are located inside the pores of the MOF, as confirmed by the characterization results demonstrated below.

CO_2_ uptakes of the as-received and activated MIL-101(Cr) were compared with the literature-reported CO_2_ uptakes (25 °C and 1 bar) on MIL-101(Cr) having different surface areas and synthesis conditions, as shown in Figure S2. The collected literature data were categorized based on the surface area and the solvent that was used for the synthesis of MIL-101(Cr). It was concluded that changes in the surface area and solvents directly affect the gas uptake of MIL-101(Cr). Nonetheless, the comparison provided in Figure S2 shows that the CO_2_ uptakes measured in this study are in the range of the literature values.^48,49^ Further comparison of experimental gas uptakes of MIL-101(Cr) with the literature are presented in Table S2.

To determine the surface area and pore volume of the MIL-101(Cr) and [MPPyr][DCA]/MIL-101(Cr) composite, N_2_ adsorption isotherms were measured. Results given in Figure S3 demonstrate that the BET surface area and the micropore volume of MIL-101(Cr) decreased from 1924 m^2^/g (having a type I isotherm based on IUPAC classification^50^) to 377 m^2^/g and from 1.001 to 0.213 cm^3^/g, respectively, upon IL incorporation as tabulated in Table S3. This decrease is expected, and it can be attributed to the blockage of the pores of MIL-101(Cr) by the IL molecules, confirming the successful incorporation of IL into the MOF pores.^29^ The N_2_ solubility in [MPPyr][DCA] was qualitatively estimated by the COSMO-RS calculations performed at the BET measurement conditions in the pressure range of 0.1 to 1 bar, at −196 °C, as shown in Figure S4. Results showed that N_2_ has almost negligible solubility in the IL at the BET measurement conditions. Hence, we infer that the IL molecules located at the pore openings of the MOFs might block the entrance of the N_2_ molecules into the available pores, making the results of the BET measurements unreliable. These observations are also consistent with previous reports presenting different functionalized porous materials.^51−54^ The CO_2_ solubilities in [MPPyr][DCA] obtained from the COSMO-RS calculations, as presented in Figure S5, were 0.0048 and 0.039 mol gas/mol IL at 0.06 bar and 0.92 bar, and at 25 °C, respectively, compared to the experimental CO_2_ solubilities of the same IL as 0.00085 and 0.012 mol gas/mol IL, respectively, under the same conditions.^55^ These results showed that the COSMO-RS results are comparable with the experimental measurements within an order of magnitude, consistent with the literature.^56^

**Figure 2:**
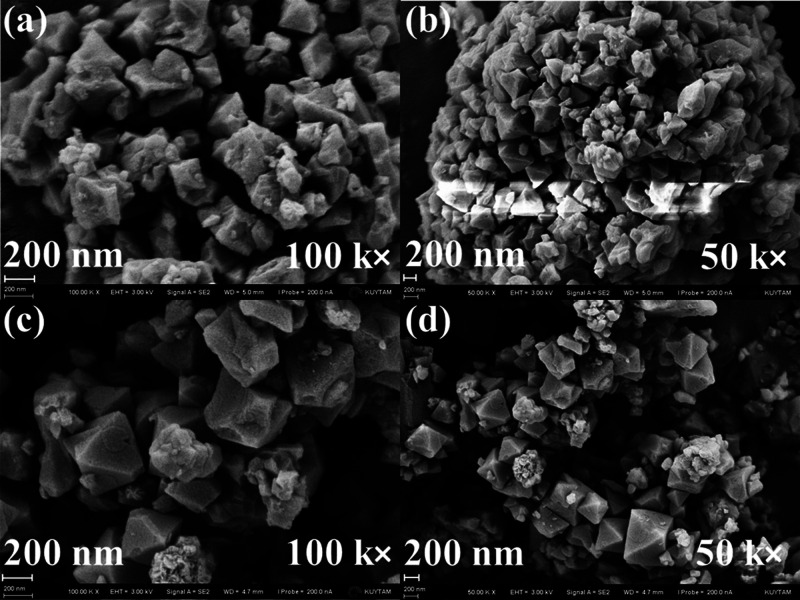
SEM images of (a,b) as-received MIL-101(Cr) and (c,d) [MPPyr][DCA]/MIL-101(Cr) composite with an IL loading of 38 wt %. Images characterizing the as-received MIL-101(Cr) given in (a) and (b) are reproduced with permission from ref (47). Copyright [2023] Elsevier.

Next, we checked the morphology of the pristine MOF and IL/MOF composite. XRD data of the pristine MIL-101(Cr) given in Figure 1b are consistent with the literature.^57,58^Figure S6 represents the XRD data of the simulated and pristine MIL-101(Cr), which showed that MIL-101(Cr) has Fd3m symmetry with diffraction peaks located at 2θ values of 8.4 for (606) and 9.0 for (753), corresponding to the main peaks of MIL-101(Cr) with a unit cell parameter of 88.3 Å, consistent with the literature.^57^ The corresponding peaks of MIL-101(Cr) are also present in [MPPyr][DCA]/MIL-101(Cr) spectra, depicting that the crystal structure of pristine MIL-101(Cr) remains intact upon IL incorporation. The [MPPyr][DCA]/MIL-101(Cr) composite was characterized by slight shifts in peaks representing (606) and (753) to lower 2θ values, while the unit cell parameter did not show any significant change. Moreover, the data showed a change in the peak intensities upon the incorporation of IL. These changes can be associated with the possible changes in the electronic structure inside the pores upon the incorporation of IL molecules.^28^ In addition, a comparison of SEM images of the as-received MIL-101(Cr) and its composite with [MPPyr][DCA] given in Figure 2 indicates that the octahedral structure of the MOF was maintained upon the incorporation of [MPPyr][DCA], consistent with the XRD results.

**Figure 3:**
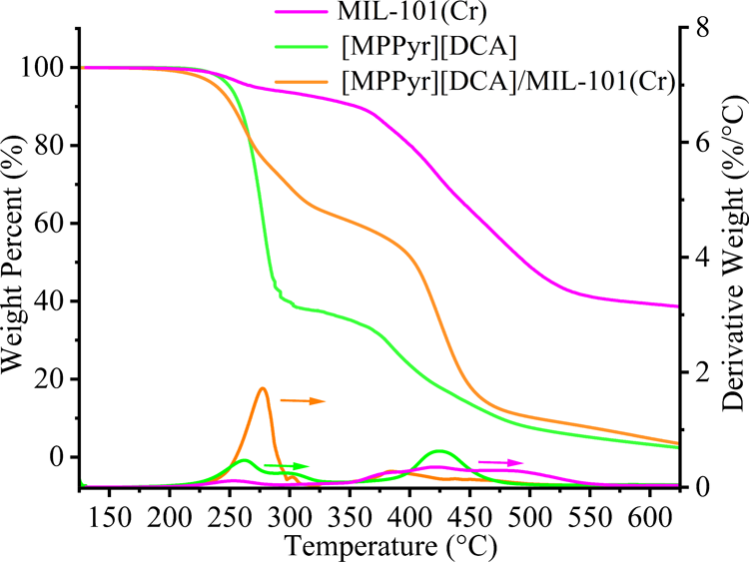
TGA and DTG curves of pristine MIL-101(Cr) and [MPPyr][DCA]/MIL-101(Cr) composites with an IL loading of 38 wt %. The data set associated with the bulk IL was reported in our previous work^47^ and provided for comparison.

The TGA measurements were conducted for the as-received MOF and its composite with [MPPyr][DCA]. The corresponding results are shown in Figure 3. The initial weight loss in a temperature range of 100 to 200 °C in each thermogravimetric curve is associated with the evaporation of residual solvent or moisture. Figure 3 indicates that MIL-101(Cr) has a *T′*_onset_ value of approximately 245 °C, which might be associated with the removal of the organic ligands.^38^ On the other hand, the *T′*_onset_ of the bulk IL was determined to be approximately 230 °C,^47^ whereas that of the IL/MOF composite was approximately 220 °C. These results indicate that the as-synthesized IL/MOF composite has a lower *T′*_onset_ compared to pristine MOF. This decrease in *T′*_onset_ was inferred to be associated with the presence of interactions between the IL molecules and the MOF cage.^38^

**Figure 4:**
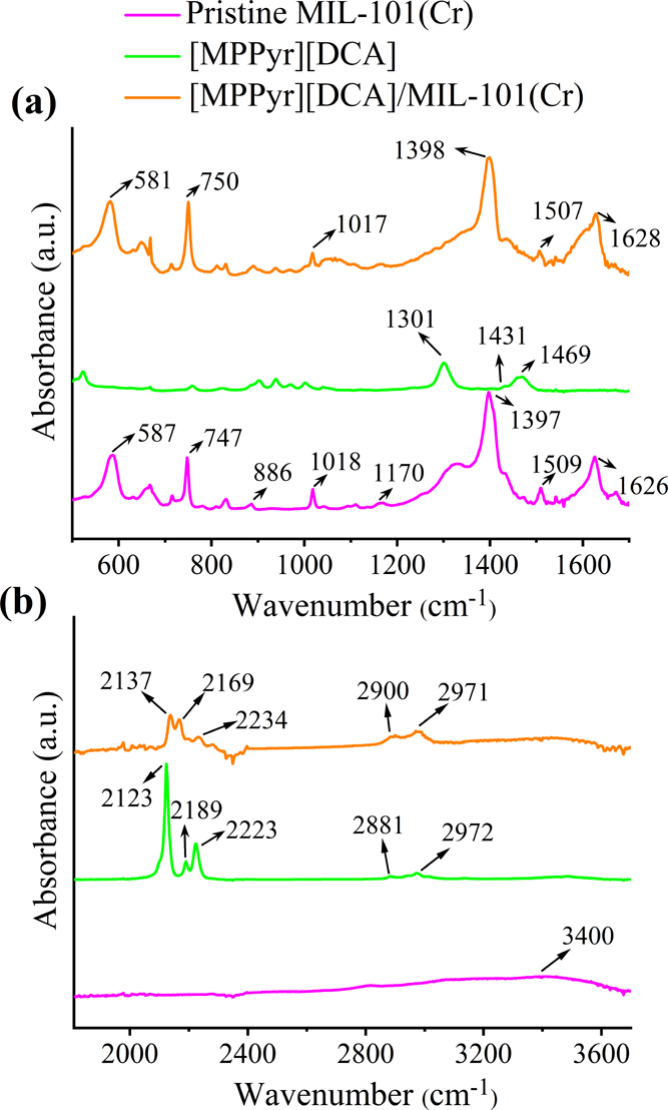
IR spectrum of the [MPPyr][DCA]/MIL-101(Cr) composite with an IL loading of 38 wt % (a) 500–1800 cm^–1^ and (b) 1800–3800 cm^–1^. The data for pristine MIL-101(Cr) and IL were taken from our previous work^47^ and provided for comparison.

To have a better understanding of these molecular IL-MOF interactions, we compared the IR spectrum of the [MPPyr][DCA]/MIL-101(Cr) composite with those of MIL-101(Cr) and the bulk [MPPyr][DCA], as shown in Figure 4a,b. The IR features at 2881 and 2972 cm^–1^ in the spectrum of the bulk [MPPyr][DCA] are assigned to the symmetric and asymmetric ν(C–H) vibrations, and those at 1301, 2123, 2189, and 2223 cm^–1^ are attributed to ν(C≡N) on [DCA]^−^.^32,59^Figure 4 demonstrates that the ν(C≡N) bands located at 2123 and 2223 cm^–1^ blue-shifted by 14 and 11 cm^–1^, respectively, when the IL molecules were incorporated into the pores. These shifts were accompanied by a blue shift of 9 cm^–1^ on the ν(C–H) peak of the IL positioned at 2881 cm^–1^.

The band at 587 cm^–1^ present in the lower frequency region of pristine MIL-101(Cr) is attributed to the coordination of the 1,4-benzene dicarboxylate (BDC) linker to the chromium nodes. Furthermore, the features present in a region from 587 to 1626 cm^–1^ are associated with the ν(C=C) and ν(C–H) of the benzene ring, positioned at 1509 cm^–1^ and 747, 886, 1018, and 1170 cm^–1^, respectively.^60^ In addition to these features, MIL-101(Cr) was also characterized with peaks located at 1397 and 1626 cm^–1^. These bands are assigned to the ν(COO)_sym_ and ν(C–O)_asym_ of the dicarboxylate groups of the linker, respectively.^61^ The data showed that ν(Cr–O) of the MOF present at 587 cm^–1^ demonstrates a stronger red shift (6 cm^–1^) than the one associated with the carboxylate group (remaining almost at the level of spectral resolution) when the IL is incorporated into the pores. These peak shifts further indicate that the interionic interactions in IL weaken as the anion interacts more with the Cr nodes of the MOF and induces possible changes in the electronic structure, as is also evident from the changes in the intensities of the characteristic features in the XRD data. Hence, we infer that the interactions between the IL and MOF are present mostly between the IL’s anion and the metal nodes.

### 3.2 Gas Adsorption and Separation Performance of [MPPyr][DCA]/MIL-101(Cr)

**Figure 5:**
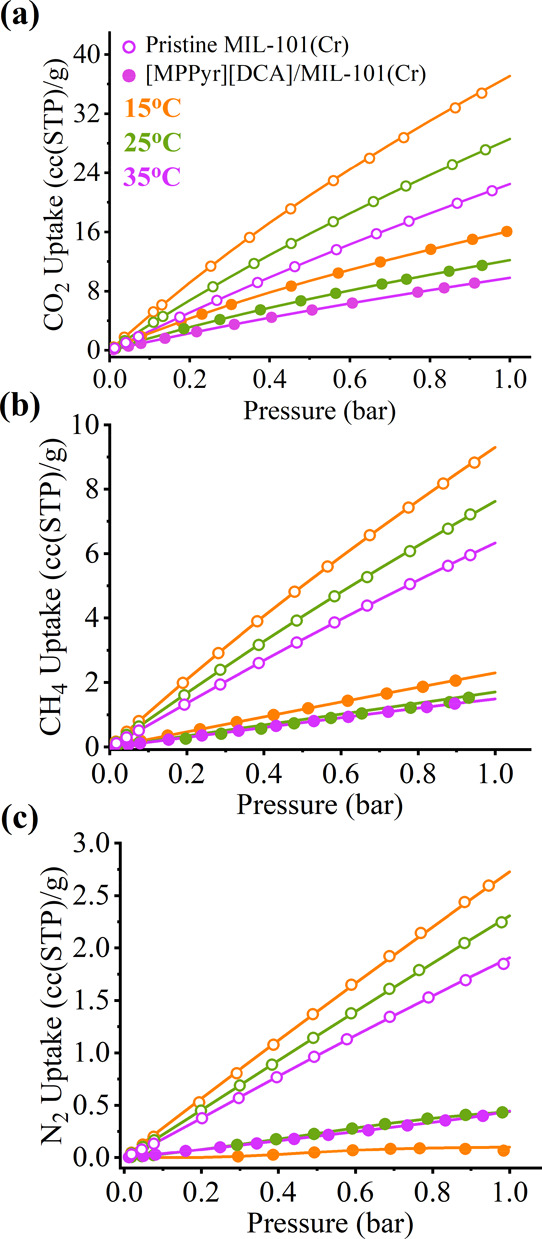
(a) CO_2_, (b) CH_4_, and (c) N_2_ adsorption uptakes of MIL-101(Cr) and its composite with [MPPyr][DCA] (having an IL loading of 38 wt %) at 15, 25, and 35 °C up to 1 bar. The empty symbols and filled symbols represent the experimental gas uptakes for pristine MIL-101(Cr) and [MPPyr][DCA]/MIL-101(Cr) composite, respectively. The lines represent the isotherms obtained by fitting the experimental data points. The data associated with the pristine MOF at 25 °C were reported in our previous work^47^ and provided for comparison.

To investigate the consequences of IL impregnation on the CO_2_/N_2_, CO_2_/CH_4_, and CH_4_/N_2_ separation performances of the MOF, single-component adsorption isotherms of CO_2_, CH_4_, and N_2_ in MIL-101(Cr) (having a type I isotherm based on IUPAC classification^50^) and its composite with [MPPyr][DCA] were measured up to 1 bar at 15, 25, and 35 °C. Results presented in Figure 5a–c and Table S4 show that CO_2_, CH_4_, and N_2_ uptakes decreased upon IL incorporation into MIL-101(Cr). These decreases in the uptake capacity of IL-incorporated MIL-101(Cr) are expected and attributed to the reduced available surface area and pore volume of the composite due to the presence of IL molecules in the pores. For instance, the composite exhibited no N_2_ adsorption (undetectable, remains below the noise level) up to 0.3 bar at 15 °C and significantly poor CH_4_ adsorption compared to the pristine MIL-101(Cr) at all temperatures, as shown in Figures 5b,c and S7. However, the CO_2_ uptakes of the composite showed a lower decrease compared to those on N_2_ and CH_4_ uptakes, which can be ascribed to the acidic nature of CO_2_ molecules having a strong affinity for [DCA]^−^, which has a highly basic nature.

We also performed COSMO-RS calculations at 15, 25, and 35 °C from 0.1 to 1 bar to investigate the temperature effect on the gas solubilities in bulk [MPPyr][DCA]. The data presented in Figure S5 demonstrate that CO_2_ has significantly higher solubility compared to CH_4_ and N_2_ at all temperatures and pressures in line with our adsorption measurements. At 1 bar and 25 °C, CO_2_ solubility in bulk [MPPyr][DCA] is 25- and 50-times higher compared to those of CH_4_ and N_2_, respectively. In addition, CO_2_ and CH_4_ solubilities slightly decrease with increasing temperature. Nonetheless, data illustrated that the N_2_ solubility in [MPPyr][DCA] was not significantly affected by temperature.

To complement the COSMO-RS results, we performed the DFT calculations. According to the results presented in Figure S8, CO_2_ comes in close contact with both ions. One of its oxygen atoms forms hydrogen bonds with the pyrrolidinium ring. On the other hand, the positively charged C atom (*q*_c_ = 1.027e) interacts with the negatively charged N atom of [DCA]^−^ (*q*_c_ = −0.606e), presenting a C–N distance of 2.81 Å. CH_4_ forms hydrogen bonds with the N atoms of [DCA]^−^, while the N_2_ molecule is forming hydrogen bonds with the pyrrolidinium ring. The binding energy calculations demonstrate that CO_2_ has the strongest interaction with [MPPyr][DCA] with a binding energy of 23.4 kJ/mol compared to 9.0 and 6.8 kJ/mol for CH_4_ and N_2_, respectively. These differences in the binding energies of the gases are also consistent with their corresponding solubilities as estimated by the COSMO-RS calculations. Therefore, we infer that relatively lower CH_4_ and N_2_ adsorption capacities compared to that of CO_2_ presented in Figure 5 are associated with their weaker interactions with the IL and hence their lower solubilities in the IL.

**Figure 6:**
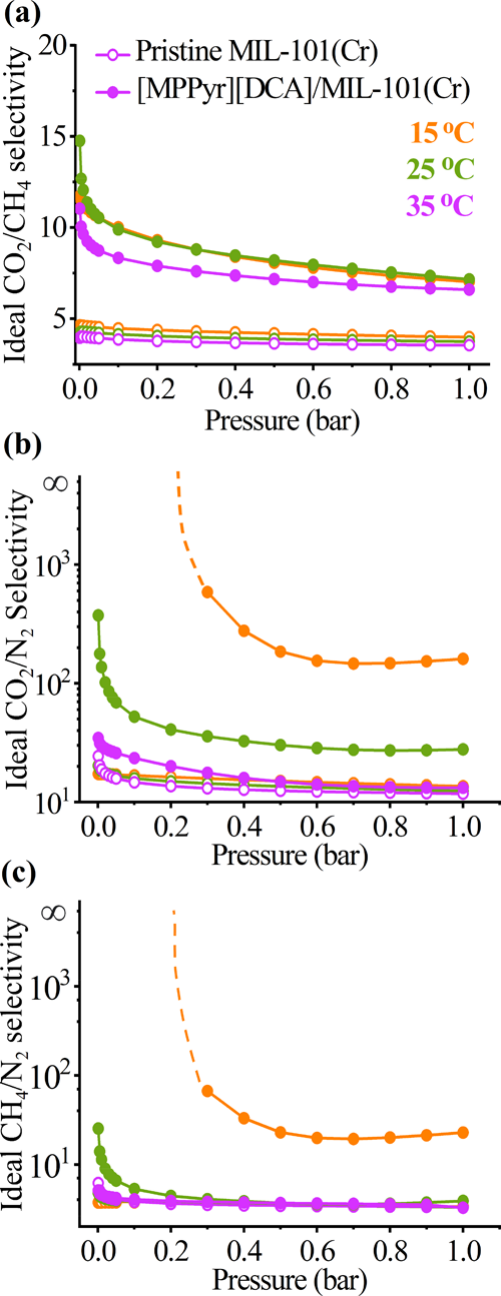
Ideal selectivities of MIL-101(Cr) and its composite with [MPPyr][DCA] at an IL loading of 38 wt % up to 1 bar and at 15, 25, and 35 °C for (a) CO_2_/CH_4_, (b) CO_2_/N_2_, and (c) CH_4_/N_2_. Ideal selectivities on the *y*-axis of (b) and (c) are given in a logarithmic scale, and the dotted lines represent practically infinite selectivity. The empty and filled symbols represent the selectivities for the pristine MOF and its composite, respectively. The data associated with the pristine MOF at 25 °C were reported in our previous work^47^ and provided for comparison.

The CO_2_ adsorption isotherm obtained at 15 °C presented in Figure 5 is fitted to the DSL model for pristine MIL-101(Cr), while all other isotherms were fitted to the DSLF model. By using these fitting parameters, the corresponding ideal selectivities of the pristine MOF and its composite with [MPPyr][DCA] were calculated up to 1 bar at 15, 25, and 35 °C, as shown in Figure 6a–c. Results showed that at low pressures, for instance, at 0.001 bar, the ideal CO_2_/N_2_, CO_2_/CH_4_, and CH_4_/N_2_ selectivities of MIL-101(Cr) were 20.2, 4.2, and 4.7 at 25 °C, respectively.^47^ As the pressure was increased to 1 bar, the ideal selectivities were slightly decreased to 12.4, 3.7, and 3.3 for CO_2_/N_2_, CO_2_/CH_4_, and CH_4_/N_2_, respectively.^47^

In the case of IL-incorporated MIL-101 (Cr), the results demonstrated a remarkable increase in the ideal selectivities for all gas pairs, as represented in Figure 6a–c. The CO_2_/N_2_ and CH_4_/N_2_ selectivities of MIL-101(Cr) boosted from 14.6 to 33.5 and from 3.7 to 4.0 at 0.1 bar at 35 °C, respectively. Moreover, the CO_2_/CH_4_ selectivity of [MPPyr][DCA]/MIL-101(Cr) was 11.1 (8.3) at 0.001 (0.1) bar corresponding to 2.7-times (2.2-times) improvement compared to pristine MIL-101(Cr). The results demonstrated that these selectivities were increased with decreasing the temperature. At 25 °C, the CO_2_/N_2_, CO_2_/CH_4_, and CH_4_/N_2_ selectivities were computed as 3535, 231.9, and 15.24 at 0.1 bar. Moreover, the CO_2_/N_2_ and CH_4_/N_2_ selectivities of MIL-101(Cr) boosted from 16.7 to 19,180 (13.6 to 160.5) and from 3.7 to 1914.9 (3.4 to 22.8) at 0.1 (1) bar at 15 °C, respectively, upon IL incorporation. We also considered the gas separation performance of the composite at 4.20 × 10^–4^ and 0.15 bar corresponding to the atmospheric and post-combustion CO_2_ capture, respectively.^63^ The CO_2_/N_2_ and CH_4_/N_2_ selectivities of MIL-101(Cr) enhanced from 14.13 to 31.30 and from 3.70 to 3.85 at 0.15 bar at 35 °C, respectively. Moreover, the CO_2_/CH_4_ selectivity of the [MPPyr][DCA]/MIL-101(Cr) composite was 9.42 (8.12) at 4.20 × 10^–4^ (0.15) bar corresponding to 2.08-times (2.12-times) improvement compared to pristine MIL-101(Cr).

Moreover, CO_2_/N_2_ and CH_4_/N_2_ selectivities of pristine MIL-101(Cr) boosted to practically infinity below 0.3 bar and from 13.6 to 160.6 and 3.4 to 22.9 at 1 bar at 15 °C, respectively, upon the incorporation of IL. In addition, at 15 °C, the CO_2_/CH_4_ selectivity of pristine MIL-101(Cr) increased from 4.60 (4.50) to 11.70 (10.0) at 0.001 (0.1) bar and 4.76 (4.45) to 13.06 (9.60) at 4.20 × 10^–4^ (0.15) bar. These increases in selectivities with the decreasing pressure are associated with the change in what drives the adsorption. The gas adsorption in the low-pressure region is governed by the interactions between the adsorbent and gas molecules, whereas at high pressures, it significantly depends on the available pore volume.^32,62^ Hence, such a remarkable boost in the selectivity of the composite in the low-pressure region can be ascribed to the change in the adsorption mechanism from pore availability to surface affinity.

Ideal selectivity is a general indication of the materials’ separation properties. However, when a process is taken into consideration, gases exist in mixtures. Therefore, we calculated the IAST selectivities to investigate the mixture separation performance of the composite. The temperature was set to 25 °C to be able to get comparable results with the literature. Figure S9 shows the mixture (CO_2_/CH_4_:50/50, CO_2_/N_2_:15/85, and CH_4_/N_2_:50/50 representing the gas concentrations associated with methane purification, flue gas separation, and natural gas upgrading, respectively) selectivities of the pristine MOF and its composite calculated using IAST. Normalized selectivity of IL/MOF composites was calculated by dividing the selectivity of IL/MOF composites by the selectivity of the pristine MOF. At 0.01 bar, CO_2_/CH_4_:50/50 mixture selectivity of pristine MIL-101(Cr) improved from 4.4 to 12.2, indicating a normalized selectivity of 2.8, as shown in Figure S9a. The normalized selectivity followed a decreasing trend as the pressure increased and became 1.8 at 1 bar. Similar trends were observed for CO_2_/N_2_ and CH_4_/N_2_ separations with normalized selectivities of 4.9 and 2.1 at 0.01 bar and 15.6 and 1.3 at 1 bar, as shown in Figure S9b,c, respectively. Moreover, it is also important to examine the CO_2_ separation performance of an adsorbent under post-combustion conditions corresponding to a pressure of 0.15 bar.^63,64^ Data indicate that the composite provides a CO_2_/N_2_ selectivity of approximately 44 at this pressure. In addition, we also note that these IAST results of the composite for CO_2_/N_2_ separation were found to be mostly comparable with those of various aminosilane-loaded UiO-67 materials under similar conditions.^65^ Hence, considering the superior gas separation performance of the composite, it is reasonable to conclude that this material has a strong potential to be used in flue gas separation and natural gas purification applications.

Table S5 compares the selectivities of the [MPPyr][DCA]/MIL-101(Cr) composite presented in this work with other IL/MOF composites reported in the literature. The ideal CO_2_/N_2_ and CH_4_/N_2_ selectivities of the [MPPyr][DCA]/MIL-101(Cr) composite presented in this study are significantly higher than those of other IL/MOF composites especially at low pressures. Such high gas separation performance can be attributed to the superior solubility of pyrrolidinium-based ILs for CO_2_.^33^ Moreover, strong interactions present between the [MPPyr][DCA] and CO_2_ molecules, as also validated by the COSMO-RS and the DFT results, facilitate the diffusion of CO_2_ molecules into the IL-incorporated MOF, while rejecting the N_2_ molecules, making it completely selective for CO_2_.

## 4 Conclusions

A new IL/MOF composite, [MPPyr][DCA]/MIL-101(Cr), with an IL loading of 38 wt %, was prepared by the wet impregnation method. The composite was characterized in detail to identify the interactions between the IL molecules and the MOF surface. IR analysis showed that the IL’s anion strongly interacts with the Cr nodes of the MOF. Moreover, DFT calculations demonstrated that CO_2_, CH_4_, and N_2_ molecules interact differently with [MPPyr][DCA]: CO_2_ interacts with both ions of [MPPyr][DCA], whereas CH_4_ and N_2_ form hydrogen bonds with the anion and cation, respectively. Gas adsorption measurements showed that the [MPPyr][DCA]/MIL-101(Cr) composite offers exceptional CO_2_ and CH_4_ selectivity over N_2_: CO_2_/N_2_ and CH_4_/N_2_ selectivities of the pristine MOF were improved to practically infinity below 0.3 bar at 15 °C upon the incorporation of the IL. It is noteworthy that the significant affinity of IL for CO_2_ and the poor solubilities of CH_4_ and N_2_ in bulk [MPPyr][DCA] result in negligible adsorption of N_2_ molecules in the composite, making it almost completely selective for CO_2_/N_2_ and CH_4_/N_2_. Overall, the CO_2_/N_2_, CH_4_/N_2_, and CO_2_/CH_4_ selectivity of the [MPPyr][DCA]/MIL-101(Cr) composite, which we measured is higher than the selectivities of imidazolium-based IL/MOF composites reported to date, showing the excellent potential of the synthesized composite.

## Supporting Information Available

IR spectra of the composite before and after washing with acetone; comparison of CO_2_ uptakes of MIL-101(Cr) samples from the literature; N_2_ physical adsorption–desorption isotherm; results of COSMO-RS calculations; simulated XRD pattern of MIL-101(Cr); gas uptakes of MIL-101(Cr) and [MPPyr][DCA]/MIL-101(Cr) at 15, 25, and 35 °C up to 0.3 bar; results of DFT calculations examining the interactions of CO_2_, CH_4_, and N_2_ with bulk [MPPyr][DCA]; IAST mixture selectivities of MIL-101(Cr) and [MPPyr][DCA]/MIL-101(Cr) composite; fitting parameters for the gas adsorption isotherms; comparison of experimental gas uptakes for pristine MIL-101(Cr) with literature; BET and Langmuir surface areas and pore volume calculation; experimental gas uptakes data for pristine MIL-101(Cr) and [MPPyr][DCA]/MIL101(Cr) composite at 15, 25, and 35 °C; and comparison of ideal selectivity of the [MPPyr][DCA]/MIL-101(Cr) composite with the literature (PDF)
