# Supplementary material for: Composite of MIL-101(Cr) with a Pyrrolidinium-Based Ionic Liquid Providing High CO2 Selectivity
Source: ACS Appl Eng Mater. 2023 May 22;1(6):1473–81. doi: 10.1021/acsaenm.3c00010 (PMC10294249; doi:10.1021/acsaenm.3c00010)
Supplement: Supplementary file 1 — em3c00010_si_001.pdf [file em3c00010_si_001.pdf]

Supporting Information

Composite of MIL-101(Cr) with a pyrrolidinium-based ionic liquid providing high CO_2 selectivity

Nitasha Habib,[a,b] Ozce Durak,[a,b,†] Hasan Can Gulbalkan,[a,†] Ahmet Safa Aydogdu,[a,b,†] Seda Keskin,[a,b,*] and Alper Uzun,[a,b,c,*]

[a]Department of Chemical and Biological Engineering Koç University, Rumelifeneri Yolu, 34450 Sariyer, Istanbul, Turkey
[b]Koç University TÜPRAŞ Energy Center (KUTEM), Koç University, Rumelifeneri Yolu, 34450 Sariyer, Istanbul, Turkey
[c]Koç University Surface Science and Technology Center (KUYTAM), Koç University, Rumelifeneri Yolu, 34450 Sariyer, Istanbul, Turkey

Submitted to ACS Applied Engineering Materials

*Corresponding authors: auzun@ku.edu.tr and skeskin@ku.edu.tr

† These authors contributed equally.

I. Figures

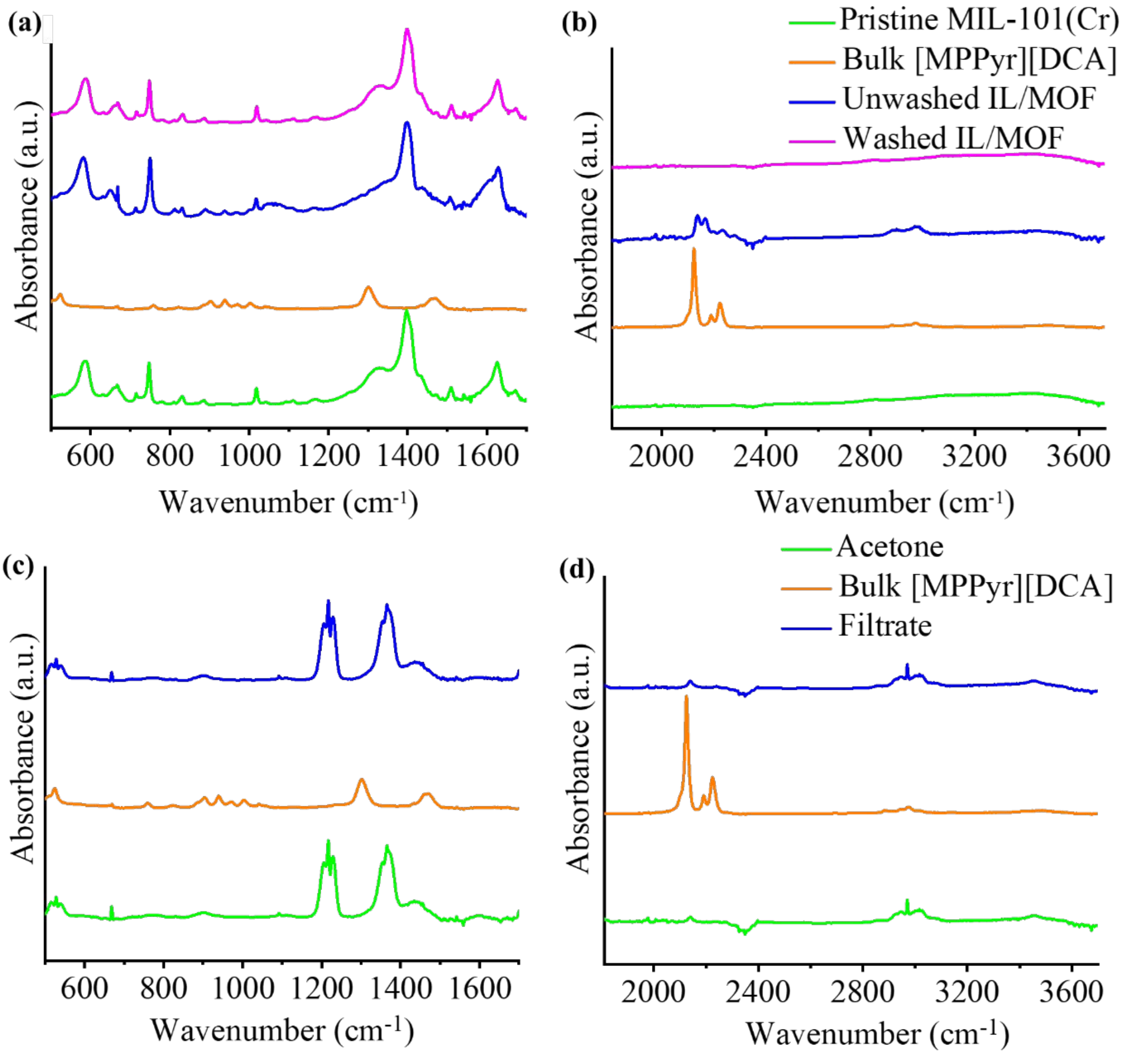

Figure S1. IR spectra of MIL-101(Cr), bulk [MPPyr][DCA], and [MPPyr][DCA]/MIL-101(Cr) before and after washing with acetone in two different regions, 600-1700 cm^{-1} and 1800-3600 cm^{-1}; Powder samples before and after washing with acetone in (a) and (b); the filtrate (liquid samples) in (c) and (d). The data set associated with pristine MIL-101(Cr) and bulk IL was reported in our previous work[1] and provided for comparison.

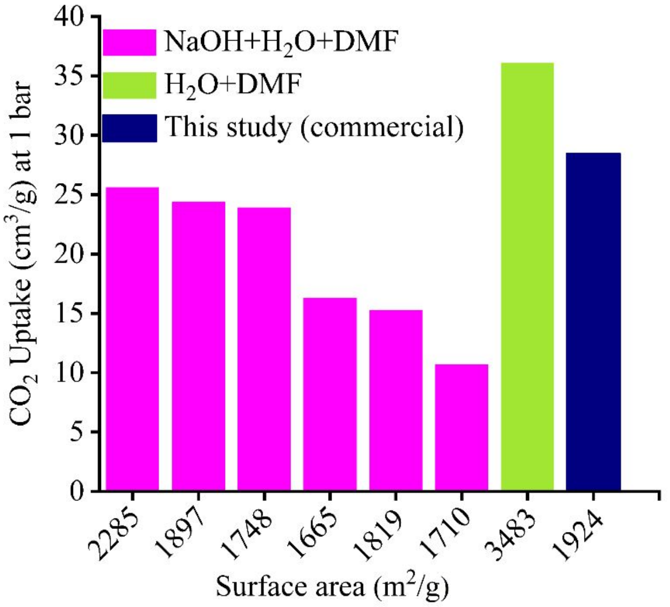

Figure S2. Comparison of CO_2 uptakes and surface areas of the different MIL-101(Cr) samples from the literature. The entries were color-coded based on the types of solvents used for the synthesis.

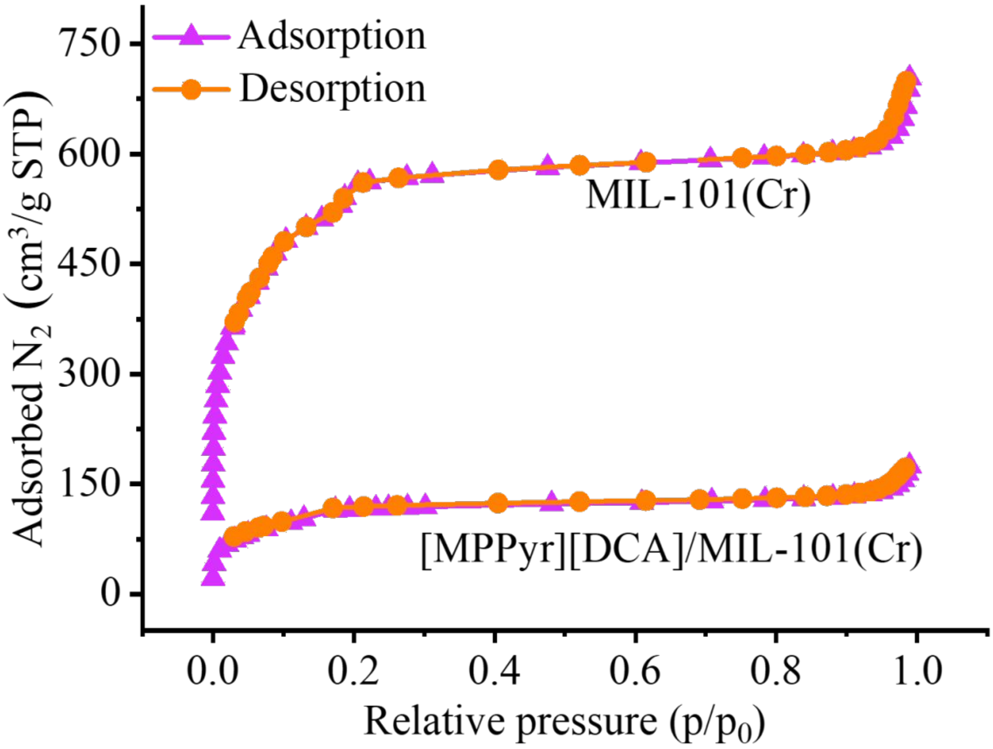

Figure S3. N_2 physical adsorption-desorption isotherm of [MPPyr][DCA]/MIL-101(Cr) composite having an IL loading of 38 wt.%. The data set associated with the pristine MIL-101(Cr) was reported in our previous work[1] and provided for comparison.

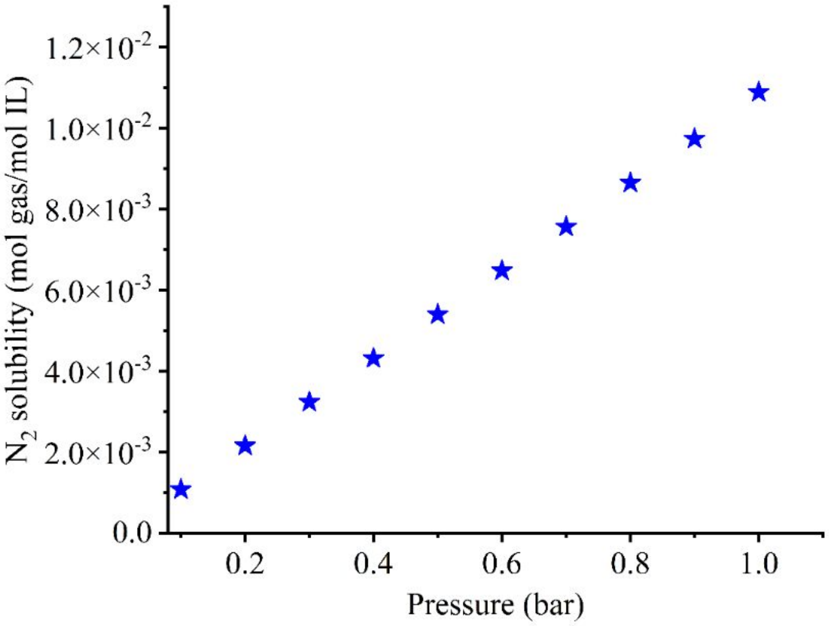

Figure S4. The solubility of N_2 in bulk [MPPyr][DCA] calculated by COSMO-RS calculations at -196 °C between 0.1-1 bar. Reproduced with permission from reference [1]. Copyright [2023] Elsevier.

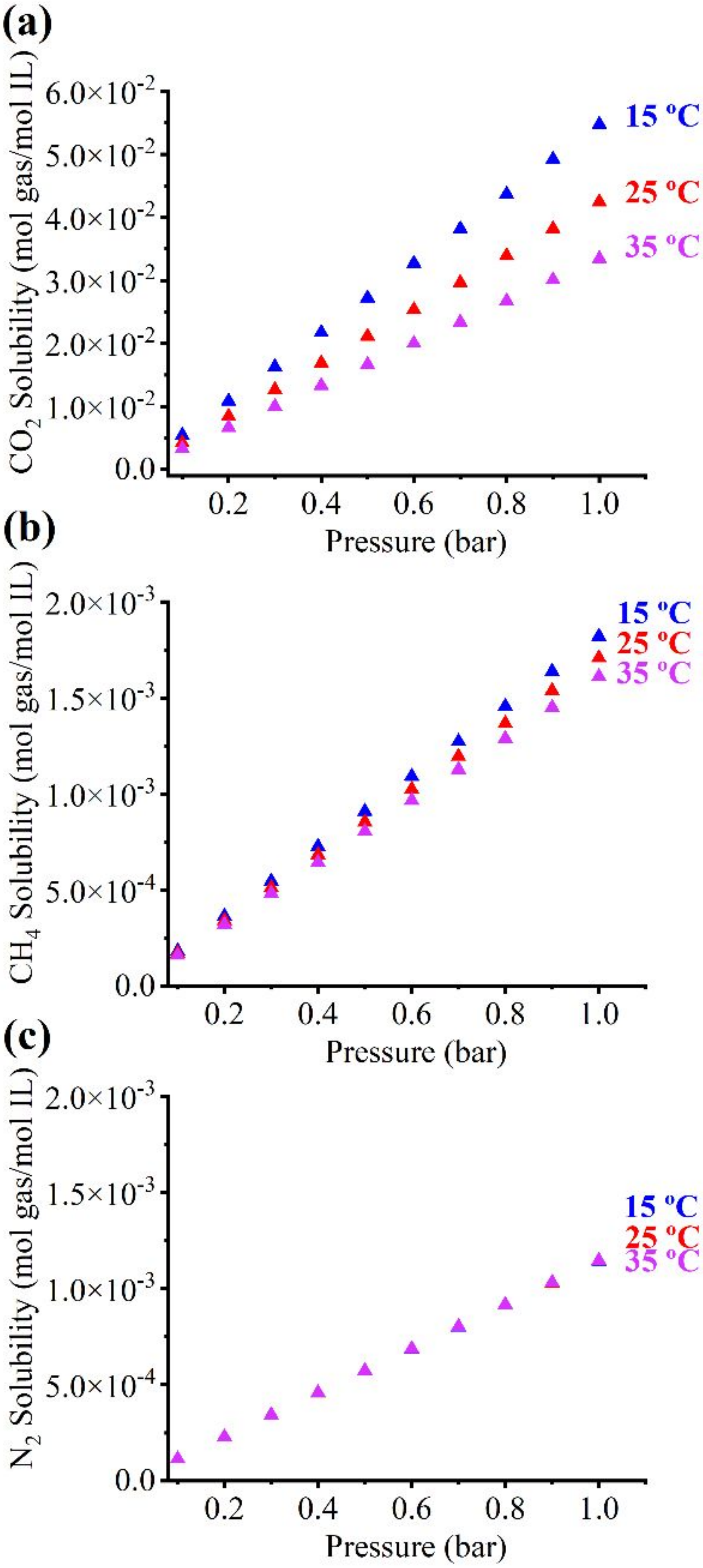

Figure S5. The solubility of (a) CO_2, (b) CH_4, and (c) N_2 in bulk [MPPyr][DCA] calculated by COSMO-RS calculations at 15, 25, and 35 °C between 0.1-1 bar. Data set associated with 25 °C were taken from our previous work[1] and used for comparison.

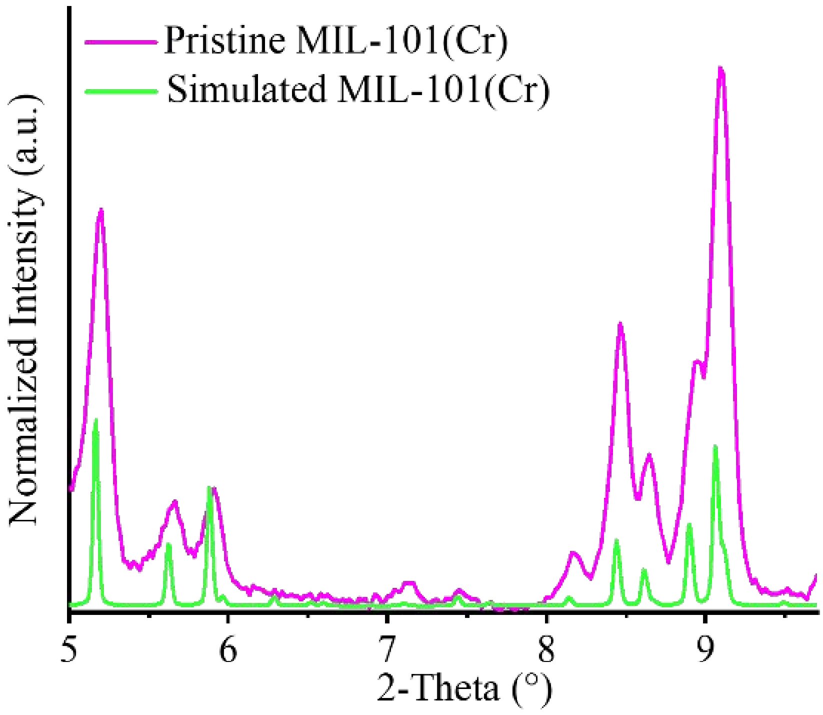

Figure S6. X-ray diffraction pattern of simulated, pristine MIL-101(Cr) in 2Θ range of 5-9.5^{0}.

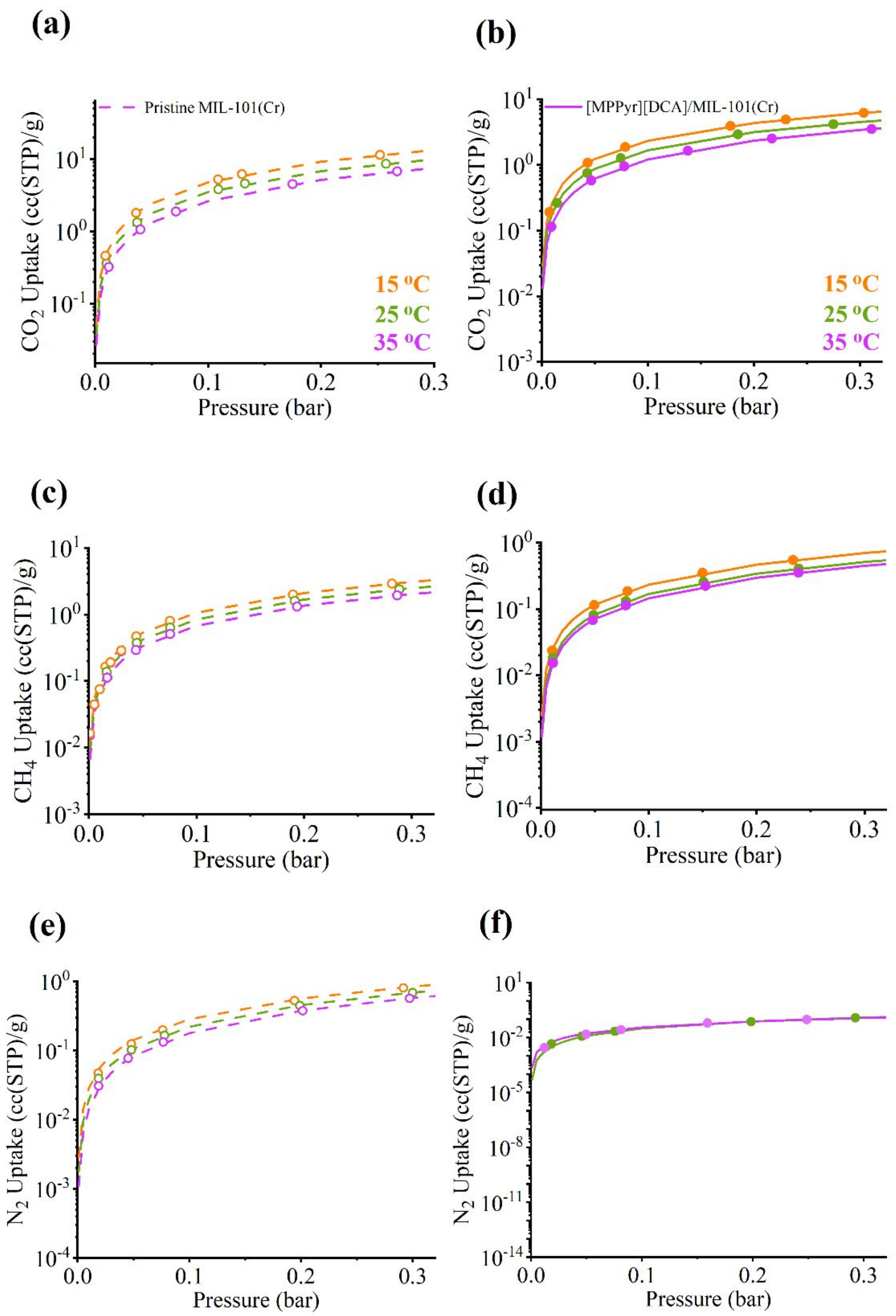

Figure S7. CO_2, CH_4, and N_2 adsorption isotherms for the pristine MIL-101(Cr) (a, c, and e, respectively) and for the [MPPyr][DCA]/MIL-101(Cr) composite (b, d, and f, respectively) at 15, 25, and 35 °C up to 0.3 bar. The circles represent the experimental data points, and the lines represent the fitted data.

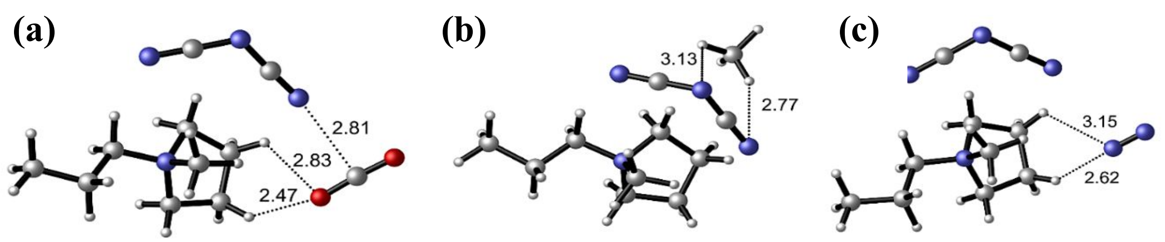

Figure S8. The equilibrium geometries representing the interactions of [MPPyr][DCA] with (a) CO_2, (b) CH_4, (c) N_2 molecules calculated at the B3LYP-D2/6-311+G* level of theory. Bond distances are given in Å. Red, grey, white, and blue spheres represent O, C, H, and N atoms, respectively.

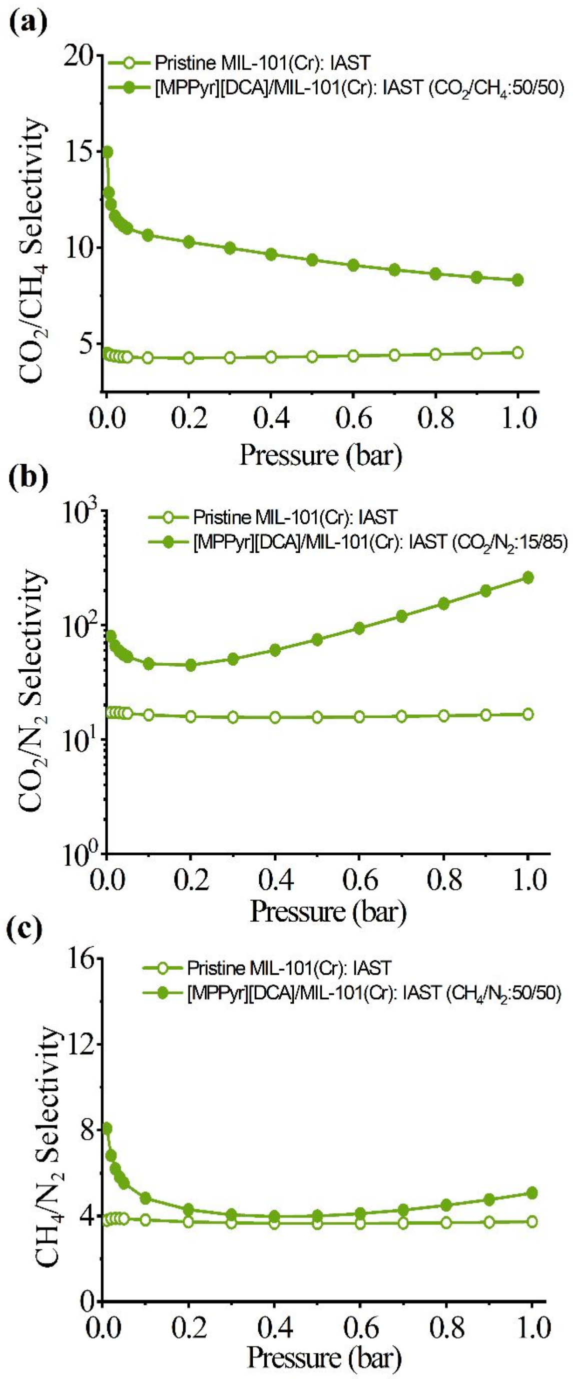

Figure S9. (a) CO_2/CH_4, (b) CO_2/N_2, (c) CH_4/N_2 IAST selectivities of MIL-101(Cr), and [MPPyr][DCA]/MIL-101(Cr) composite up to 1 bar and at 25 °C. The empty symbols and filled symbols represent selectivities for pristine MIL-101(Cr) and [MPPyr][DCA]/MIL-101(Cr) composite, respectively.

II. Tables

Isotherms were fitted to Dual-site Langmuir-Freundlich (DSLF) model and Dual-site Langmuir (DSL) model as given in the following equations:

$$\text{DSLF} \rightarrow n(P) = q_1 \times ((k_1 \times P)^{n_1}/(1 + k_1 \times P)^{n_1}) + q_2 \times ((k_2 \times P)^{n_2}/(1 + k_2 \times P)^{n_2}).$$

$$\text{DSL} \rightarrow n(P) = q_1 \times ((k_1 \times P)/(1 + k_1 \times P)) + q_2 \times ((k_2 \times P)/(1 + k_2 \times P)).$$

Here, n(P) represents adsorption quantity under equilibrium temperature, and P represents pressure. q_1 and q_2 represent the maximum adsorption capacity on sites #1 and #2, while k_1 and k_2 are the temperature-dependent equilibrium constants. n_1 and n_2 are the corresponding fitting parameters.

Table S1. Dual-site Langmuir-Freundlich fit parameter for pristine in MIL-101(Cr) and [MPPyr][DCA]/MIL 101(Cr) composite with an IL loading of 38 wt.%.

	**Dual-Site Langmuir Model**					
	**Gas**	**q_1 (cc/g)**	**k_1 (1/mbar)**	**q_2 (cc/g)**	**k_2 (1/mbar)**	**R^2**
MIL-101(Cr) 15 °C	CO_2	244.82	0.13	14.43	1.21	1
	N_2	30.68	0.01	118.71	0.01	0.99

	**Dual-site Langmuir-Freundlich Model**							
	**Gas**	**q_1 (cc/g)**	**k_1 (1/mbar)**	**n_1***	**q_2 (cc/g)**	**k_2 (1/mbar)**	**n_2***	**R^2**
MIL-101(Cr) 15 °C	CH_4	43.54	0.16	1.46	18.22	0.53	0.98	0.99
MIL-101(Cr)[1] 25 °C	CO_2	7.6×10^{-15}	1.7×10^{-17}	4.1×10^{-7}	157.6	0.21	0.99	0.99
	N_2	2.55	0.17	0.82	9.30	0.31	1.23	0.99
	CH_4	33.77	0.16	1.31	16.51	0.40	0.97	0.99
MIL-101(Cr) 35 °C	CO_2	70.65	0.25	1.50	35.42	0.66	0.97	0.99
	N_2	12.70	0.20	1.09	0.002	0.006	1.48×10^{-11}	0.99
	CH_4	30.22	0.19	1.23	8.52	0.46	0.94	0.99
[MPPyr] [DCA]/MIL-101(Cr) 15 °C	CO_2	80.25	0.19	1.03	5.17	2.05	0.93	0.99
	N_2	0.005	0.009	51.26	0.10	1.95	4.15	0.99
	CH_4	55.17	0.33	5.51	14.1	0.19	1.04	0.99
[MPPyr] [DCA]/MIL-101(Cr)	CO_2	73.10	0.13	1.20	12.65	0.96	0.94	0.99
	N_2	0.16	1.43	3.75	0.47	1.52	1.40	0.99

25 °C	CH_4	4.21	0.48	1.05	12.17	0.21	2.28	0.99
[MPPyr] [DCA]/MIL-101(Cr) 35 °C	CO_2	11.04	1.09	0.99	22.01	0.40	1.64	0.99
	N_2	4.01	0.79	23.03	14.31	0.04	1.08	0.99
	CH_4	1.73	0.58	2.02	2.97	0.55	1.04	0.99

Table S2. Comparison of experimental CO_2, CH_4, and N_2 uptakes for pristine MIL-101(Cr) reported in the literature.

Reference	Pressure (bar)	Temperature (°C)	Experimental Uptakes (cm^3/g)		
			CO_2	CH_4	N_2
This study	0.001	15	0.049	0.0106	0.0028
		25[1]	0.036	0.0085	0.0018
		35	0.027	0.0068	0.0011
	0.01	15	0.491	0.106	0.028
		25[1]	0.359	0.083	0.020
		35	0.267	0.067	0.014
	0.1	15	4.754	1.062	0.283
		25[1]	3.492	0.841	0.220
		35	2.614	0.676	0.178
	1	15	37.103	9.298	2.727
		25[1]	28.601	7.623	2.307
		35	22.483	6.331	1.908
2	0.01	25	0.397		
		35	0.246		
	1	25	20.243		
		35	23.744		
3	0.01	25	0.448		
	1	25	36.064		
4	0.1	15	16.912	0.941	0.163
		30	5.488	0.587	0.105
		40	4.704	0.381	0.093
	1	15	53.760	8.960	4.928
		30	35.840	5.712	2.815
		40	35.280	5.152	2.778

Table S3. BET and Langmuir surface areas and pore volumes of pristine MIL-101(Cr) and [MPPyr][DCA]/MIL-101(Cr) having an IL loading of 38 wt.%.

Sample	S_{BET} (m^2/g)	$S_{Langmuir}$ (m^2/g)	$V_{Micropore}$ (cm^3/g)[a]	$V_{Micropore}$ (cm^3/g)[b]	V_{total} (cm^3/g)
MIL-101(Cr)	1924	2969	1.001	0.488	1.1574
[MPPyr][DCA]/MIL-101(Cr)	377	591	0.213	0.167	0.2702

[a]Based on the Dubinin-Astakhov equation
[b]Based on the t-plot

Table S4. CO_2, CH_4, and N_2 uptakes data for pristine MIL-101(Cr) and [MPPyr][DCA]/MIL-101(Cr) composite at 15, 25, and 35 °C.

[MPPyr][DCA]/MIL-101(Cr)					
**15 °C**					
N_2		CO_2		CH_4	
Pressure (bar)	Uptake (cc/g)	Pressure (bar)	Uptake (cc/g)	Pressure (bar)	Uptake (cc/g)
0.018	-0.004	0.007	0.191	0.011	0.024
0.047	-0.007	0.043	1.080	0.050	0.115
0.077	-0.010	0.079	1.874	0.080	0.186
0.200	-0.002	0.178	3.915	0.150	0.352
0.295	0.010	0.230	4.902	0.234	0.547
0.386	0.026	0.303	6.211	0.329	0.768
0.493	0.049	0.454	8.683	0.425	0.991
0.593	0.069	0.571	10.451	0.515	1.200
0.691	0.083	0.676	11.955	0.617	1.432
0.766	0.088	0.802	13.656	0.718	1.656
0.885	0.082	0.907	15.007	0.812	1.864
0.984	0.066	0.993	16.075	0.898	2.057
**25 °C**					
N_2		CO_2		CH_4	
Pressure (bar)	Uptake (cc/g)	Pressure (bar)	Uptake (cc/g)	Pressure (bar)	Uptake (cc/g)
0.018	0.005	0.015	0.264	0.019	0.018
0.046	0.012	0.043	0.755	0.045	0.081
0.076	0.022	0.075	1.269	0.076	0.131
0.198	0.075	0.185	2.931	0.197	0.257
0.293	0.122	0.275	4.170	0.289	0.408
0.394	0.176	0.377	5.486	0.393	0.567
0.492	0.226	0.478	6.711	0.478	0.732
0.591	0.277	0.565	7.717	0.574	0.897
0.676	0.321	0.680	8.977	0.654	1.040
0.786	0.372	0.741	9.627	0.780	1.215
0.886	0.407	0.848	10.699	0.881	1.390
0.981	0.432	0.931	11.490	0.932	1.524
**35 °C**					
N_2		CO_2		CH_4	
Pressure (bar)	Uptake (cc/g)	Pressure (bar)	Uptake (cc/g)	Pressure (bar)	Uptake (cc/g)
0.012	0.003	0.009	0.114	0.011	0.015
0.050	0.015	0.047	0.579	0.048	0.068
0.081	0.027	0.078	0.951	0.079	0.114
0.159	0.064	0.138	1.648	0.152	0.224
0.249	0.098	0.217	2.527	0.239	0.355
0.344	0.135	0.311	3.518	0.334	0.501
0.441	0.179	0.404	4.472	0.432	0.652

0.530	0.217	0.506	5.458	0.530	0.801
0.633	0.261	0.606	6.403	0.619	0.933
0.735	0.308	0.769	7.865	0.722	1.089
0.833	0.355	0.835	8.453	0.822	1.236
0.931	0.398	0.912	9.102	0.895	1.344

Pristine MIL-101(Cr)

15 °C

N_2		CO_2		CH_4	
Pressure (bar)	Uptake (cc/g)	Pressure (bar)	Uptake (cc/g)	Pressure (bar)	Uptake (cc/g)
0.018	0.047	0.009	0.459	0.015	0.164
0.048	0.125	0.036	1.795	0.044	0.469
0.076	0.199	0.130	6.159	0.075	0.802
0.194	0.528	0.109	5.221	0.189	1.988
0.292	0.805	0.252	11.380	0.282	2.912
0.387	1.077	0.349	15.257	0.383	3.899
0.489	1.369	0.452	19.122	0.479	4.814
0.589	1.650	0.559	22.932	0.565	5.598
0.688	1.922	0.649	25.989	0.674	6.571
0.769	2.143	0.734	28.775	0.775	7.429
0.883	2.439	0.864	32.801	0.865	8.174
0.946	2.595	0.930	34.785	0.946	8.822

25 °C

N_2		CO_2		CH_4	
Pressure (bar)	Uptake (cc/g)	Pressure (bar)	Uptake (cc/g)	Pressure (bar)	Uptake (cc/g)
0.019	0.040	0.010	0.359	0.016	0.138
0.048	0.102	0.037	1.323	0.044	0.374
0.078	0.166	0.133	4.585	0.075	0.634
0.199	0.448	0.109	3.806	0.191	1.601
0.300	0.689	0.257	8.597	0.288	2.393
0.383	0.887	0.361	11.744	0.385	3.165
0.490	1.143	0.453	14.448	0.482	3.920
0.589	1.377	0.559	17.393	0.582	4.674
0.688	1.610	0.659	20.100	0.662	5.269
0.765	1.789	0.740	22.209	0.774	6.073
0.883	2.047	0.856	25.117	0.875	6.770
0.979	2.245	0.939	27.137	0.942	7.216

35 °C

N_2		CO_2		CH_4	
Pressure (bar)	Uptake (cc/g)	Pressure (bar)	Uptake (cc/g)	Pressure (bar)	Uptake (cc/g)
0.019	0.031	0.012	0.320	0.017	0.113
0.045	0.077	0.040	1.058	0.044	0.293
0.077	0.133	0.071	1.880	0.075	0.509
0.201	0.376	0.175	4.500	0.193	1.311
0.297	0.569	0.267	6.766	0.286	1.936
0.396	0.767	0.369	9.181	0.387	2.601
0.493	0.962	0.462	11.312	0.485	3.240
0.578	1.130	0.566	13.613	0.583	3.862
0.689	1.344	0.666	15.759	0.668	4.385
0.789	1.528	0.748	17.449	0.779	5.051
0.886	1.693	0.869	19.881	0.877	5.621
0.984	1.848	0.956	21.576	0.936	5.950

Table S5. Comparison of IL-incorporated MOF composites reported in the literature for adsorption-based gas separation applications.

IL/MOF composites	Operating conditions (Pressure, Temperature)	Ideal Selectivities			IAST Selectivities			Ref.
		CO_2/CH_4	CO_2/N_2	CH_4/N_2	CO_2/CH_4 (50/50)	CO_2/N_2 (15/85)	CH_4/N_2 (50/50)	
Pristine MIL-101(Cr)	0.001 bar, 25 °C	5.8[1]	20.62[1]	3.5[1]	4.5	17.2	3.8	This study
	0.01 bar, 25 °C	5.8[1]	19.4[1]	3.3[1]	4.5	17.2	3.8	
	1 bar, 25 °C	4.7[1]	13.9[1]	2.9[1]	4.5	16.6	3.8	
[MPPyr][DCA]/MIL-101(Cr) with an IL loading of 38 wt.%	0.001 bar, 25 °C	14.8	374.5	25.4	14.9	163.7	14.3	This study
	0.01 bar, 25 °C	12.1	137.1	11.4	12.2	79.9	4.8	
	1 bar, 25 °C	7.2	27.8	3.9	8.3	260.0	5.0	
Pristine ZIF-8	0.01 bar, 25 °C	2.4	7.8	2.8	2.4	7.8	2.8	[5]
	0.1 bar, 25 °C	2.4	7.8	2.8	2.4	7.8	2.8	[5]
[BMIM][PF_6]/ZIF-8	0.1 bar, 25 °C	8.9	24.2	2.8	8.9	28.0	2.7	[5]
[BMIM][BF_4]/ZIF-8	0.1 bar, 25 °C	4.3	14.6	3.4				[6]
[BMIM][SCN]/ZIF-8	0.01 bar, 25 °C	6.5	21.2		6.5	20.9		[7]
[BMIM][$MeSO_3$]/ZIF-8	0.01 bar, 25 °C	1.7	19.1	11.3				[8]
[BMIM][$OcSO_4$]/ZIF-8	0.01 bar, 25 °C	4.7	2.6	0.53				[8]
[BMIM][$OcSO_4$]/ZIF-8	1 bar, 25 °C	3.6	6.7	1.9				[8]
Pristine CuBTC	0.01 bar, 25 °C	4.6	17.6	3.7		19.0	3.5	[9]
[BMMIM][PF_6]/ CuBTC	0.01 bar, 25 °C		23.1	4.4		21.6	4.6	[9]
[BMIM][PF_6]/ CuBTC	0.01 bar, 25 °C		26.1	5.2		26.5	5.2	[9]
[EMIM][DEP]/CuBTC	0.01 bar, 25 °C	7.3	42.3		6.6	31.1		[10]
[BMIM][NTF_2]/CuBTC	0.01 bar, 25 °C	3.2	13.1	3.8				[11]
[BMIM][CF_3SO_3]/CuBTC	0.01 bar, 25 °C	4.1	19.4	4.5				[11]
[BMIM][BF_4]/CuBTC	0.01 bar, 25 °C	4.6	21.1	4.7				[11]
[BMIM][$MeSO_4$]/CuBTC	0.01 bar, 25 °C	4.4	21.8	4.9				[11]
[BMIM][SbF_6]/CuBTC	0.01 bar, 25 °C	5.5	25.7	4.4				[11]
[BMIM][$OcSO_4$]/CuBTC	0.01 bar, 25 °C	5.4	26.7	5.0				[11]
[BMIM][$MeSO_3$]/CuBTC	0.01 bar, 25 °C	5.6	27.1	4.8				[11]
[BMIM][SCN]/CuBTC	0.01 bar, 25 °C	5.7	29.6	5.2				[11]

III. References

1. Habib, N.; Durak, Ö.; Uzun, A.; Keskin, S., Incorporation of a Pyrrolidinium-Based Ionic Liquid/MIL-101 (Cr) Composite into Pebax Sets a New Benchmark for CO_2/N_2 Selectivity. *Sep. Purif. Technol.* **2023,** *312*, 123346.
2. Soltanolkottabi, F.; Talaie, M. R.; Aghamiri, S.; Tangestaninejad, S., Introducing a Dual-Step Procedure Comprising Microwave and Electrical Heating Stages for the Morphology-Controlled Synthesis of Chromium-Benzene Dicarboxylate, MIL-101 (Cr), Applicable for CO_2 Adsorption. *J. Environ. Manage.* **2019,** *250*, 109416.
3. Zhong, R.; Yu, X.; Meng, W.; Liu, J.; Zhi, C.; Zou, R., Amine-Grafted MIL-101 (Cr) via Double-Solvent Incorporation for Synergistic Enhancement of CO_2 Uptake and Selectivity. *ACS Sustainable Chem. Eng.* **2018,** *6* (12), 16493-16502.
4. Munusamy, K.; Sethia, G.; Patil, D. V.; Rallapalli, P. B. S.; Somani, R. S.; Bajaj, H. C., Sorption of Carbon Dioxide, Methane, Nitrogen and Carbon Monoxide on MIL-101 (Cr): Volumetric Measurements and Dynamic Adsorption Studies. *Chem. Eng. J.* **2012,** *195*, 359-368.
5. Kinik, F. P.; Altintas, C.; Balci, V.; Koyuturk, B.; Uzun, A.; Keskin, S., [BMIM][PF_6] Incorporation Doubles CO_2 Selectivity of ZIF-8: Elucidation of Interactions and Their Consequences on Performance. *ACS Appl. Mater. Interfaces* **2016,** *8* (45), 30992-31005.
6. Koyuturk, B.; Altintas, C.; Kinik, F. P.; Keskin, S.; Uzun, A., Improving Gas Separation Performance of ZIF-8 by [BMIM][BF_4] Incorporation: Interactions and Their Consequences on Performance. *J. Phys. Chem. C* **2017,** *121* (19), 10370-10381.
7. Zeeshan, M.; Keskin, S.; Uzun, A., Enhancing CO_2/CH_4 and CO_2/N_2 Separation Performances of ZIF-8 by Post-Synthesis Modification with [BMIM][SCN]. *Polyhedron* **2018,** *155*, 485-492.
8. Zeeshan, M.; Kulak, H.; Kavak, S.; Polat, H. M.; Durak, O.; Keskin, S.; Uzun, A., Influence of Anion Size and Electronic Structure on the Gas Separation Performance of Ionic Liquid/ZIF-8 Composites. *Microporous Mesoporous Mater.* **2020,** *306*, 110446.
9. Nozari, V.; Zeeshan, M.; Keskin, S.; Uzun, A., Effect of Methylation of Ionic Liquids on the Gas Separation Performance of Ionic Liquid/Metal–Organic Framework Composites. *CrystEngComm* **2018,** *20* (44), 7137-7143.
10. Zeeshan, M.; Gulbalkan, H. C.; Haslak, Z. P.; Keskin, S.; Uzun, A., Doubling CO_2/N_2 Separation Performance of CuBTC by Incorporation of 1-n-Ethyl-3-Methylimidazolium Diethyl Phosphate. *Microporous Mesoporous Mater.* **2021,** *316*, 110947.
11. Nozari, V.; Keskin, S.; Uzun, A., Toward Rational Design of Ionic Liquid/Metal–Organic Framework Composites: Effects of Interionic Interaction Energy. *ACS Omega* **2017,** *2* (10), 6613-6618.
